# Darwinism on the Stage

**Published:** 1889-12-07

**Authors:** 


### Darwinism on the Stage.

M. Alphonse Daudet, the popular French novelist—whom Mr. Saintsbury, with a levity that does his critical faculty no credit, persists in regarding as a kind of Gallic Dickens—has recently joined the ranks of the philosophers; but, so far as his philosophy has yet manifested itself, we are inclined to think, *révérence parler*, that it belongs to that "school" of "philosophers" whose qualities Carlyle was in the habit of summing up in two very emphatic little words. About fifteen months ago he astonished his best friends, even those who were well aware what freaks the Meridional temperament was capable of, by exploding in *L'Immortel*, into an at ack on the French Academy, which was as violent and venomous as it was unfair and useless. And a few days ago he produced on the French stage a play called "*La Lutte pour la Vie*," in which he has thought fit to attack "*le grand Darwin*" by allusion and implication, and to make him responsible for a large part of the French crime of the day. The play has met with a certain amount of success in Paris; and, as it seems to be a "law" of our nature that we (*nous autres Anglais*) should supplement any deficiencies in our own inclination to folly by imitating our French friends, it is to be reproduced as speedily as possible on the English stage. Miss Genevieve Ward, we are authoritatively informed, "has acquired the rights in '*La Lutte pour la Vie*,'" and will shortly appear in it, "supported by Mr. George Alexander." In order the better to prepare our minds for this event, M. Daudet published his play on Sunday last, and prefixed to it a preface in which, with rather an imposing expenditure of verbiage, he tells us, in a manner that reminds us somewhat too funnily of his "*tambourinaire*" in "*Numa Roumestan*," how "the high thought" of his "work" and "its vast title" occurred to him. Under ordinary circumstances the mind of the French novelist and playwright of the present day is, perhaps, better left to revolve in its own orbit, undisturbed by English criticism; but when, as in M. Daudet's case, he threatens to invade England, and his influence will soon be pervading a large number of the more light-headed members of our community, it seems to be time to examine what there is in that mind—more especially as it professes to be informed by a strong moral motive. It would appear, then—to judge from the "foot" of this Gallic Hercules—that M. Daudet thinks that the theories of Darwin, when applied, "are infamous because they seek out the brute which is at the core of man and awake what remains on all fours in the risen quadruped"; and he declares that there is "a new race of *petits féroces* in French society (who were unknown before the Franco-German War), to whom the Darwinian formula of the 'struggle for life' serves as the pretext and excuse for every kind of shabby trick and infamy." His attention was first drawn to the existence of these obnoxious monsters by the trial of two abnormal wretches of the criminal class who based the plea for their crime "on the Darwinian theories," and one of whom delivered a lecture on the subject. And so impressed was M. Daudet by this incident, "so true" did he feel it to be, and "so contemporary," that he actually began a book about it, "half romance, half history," and "worked at it for some months," until . . . . someone else published a novel which happened to be "exactly the book that he wished to write!" He thereupon renounced his own work; but "*le struggle for lifeur*" (as he calls him) continued to "haunt" him so persistently, and he met him so frequently in literary, political, and social circles that, at last—like Thackeray, when he was in travail with "The Book of Snobs"—he could stand it no longer; and the result was: firstly, *L'Immortel;* and secondly, "*La Lutte pour la Vie.*" Now, had we not an insuperable objection to "calling names," we should certainly characterise M. Daudet's preface as a new way of talking downright nonsense; but we prefer to leave it, as Bacon did his name and memory, "to men's charitable speeches," reserving to ourselves the right of stronger speech in the event of *the struggle for lifeur* becoming more aggressive; for, however earnestly we may endeavour, by our strictures, to mitigate the follies of the day, we do not therefore hold ourselves aloof from our fellow blunderers. On the contrary, we feel, with George Eliot, that "if the human race has a bad reputation," we cannot, in spite of the editorial We, "escape being compromised."

